# The Novel Ulnar Nerve Coverage Method Which Has the Potential to Prevent the Postoperative Ulnar Neuropathy after Plate Fixation of Distal Humerus Fractures: Three Case Reports

**DOI:** 10.1155/2023/5008141

**Published:** 2023-11-27

**Authors:** Hiroki Shibayama, Shintaro Yamaga, Yutaro Sugawara, Tsuyoshi Asano, Norimasa Iwasaki

**Affiliations:** ^1^Department of Orthopaedic Surgery, KKR Sapporo Medical Center, 6-3-40, Hiragishi 1-jo, Toyohira-ku, Sapporo, Hokkaido 062-0931, Japan; ^2^Department of Orthopaedic Surgery, Faculty of Medicine and Graduate School of Medicine, Hokkaido University, Nishi 7, Kita 15, Kita-ku, Sapporo, Hokkaido 060-8638, Japan

## Abstract

**Introduction:**

Postoperative ulnar neuropathy is still an unresolved complication in patients undergoing plate fixation of distal humerus fractures. We hypothesized that decreased blood flow to the ulnar nerve due to intraoperative procedures is an important factor in the development of postoperative ulnar neuropathy. We herein report three cases of distal humerus fractures in which the soft tissues surrounding the ulnar nerve were preserved as much as possible and finally not transferred anteriorly. *Case Presentation*. A 76-year-old woman, 82-year-old woman, and 34-year-old woman underwent plate fixation for distal humerus fractures. None of the patients developed postoperative ulnar neuropathy, and there were no complaints of numbness after postoperative day 1. Nerve conduction studies were performed after 3 months postoperatively and revealed that the motor nerve conduction velocities and compound motor nerve action potentials of the ulnar nerve in two of the three patients were higher than those of the noninjured side. In one of the three patients, these values were slightly lower than those of the noninjured side. All three patients achieved bony union after several months postoperatively.

**Conclusions:**

We obtained good outcomes with the ulnar nerve coverage method for preventing postoperative ulnar neuropathy in patients with distal humerus fractures. Preservation of blood flow to the ulnar nerve was considered important, and anatomical repositioning of the ulnar nerve after plate fixation has the potential to prevent adhesion between the ulnar nerve and the plate.

## 1. Introduction

Postoperative ulnar neuropathy is still an unresolved complication in patients undergoing plate fixation of distal humerus fractures. A meta-analysis in 2018 showed that the incidence of this complication is not low (19.3%) [1]. It is an important problem for clinicians to overcome because of the risk of permanent disability.

Methods to prevent postoperative ulnar neuropathy have not yet been established. Although anterior transposition is often used to prevent neuropathy, a previous meta-analysis showed no significant association between the performance of anterior transposition and the incidence of ulnar neuropathy [1]. Other authors reported no preventive effect of anterior transposition but instead found that anterior transposition was associated with a higher incidence of ulnar neuropathy [2]. These findings suggest that another factor, different from the presence or absence of anterior transposition, may be involved in the development of postoperative ulnar neuropathy.

We hypothesized that decreased blood flow to the ulnar nerve due to intraoperative procedures is an important factor in the development of postoperative ulnar neuropathy. In 2017, Moritani et al. [3] reported a minimally invasive ulnar nerve transposition technique in which the ulnar nerve is dissected with the surrounding soft tissues, such as the triceps muscle and periosteum. We have modified this technique and named it the ulnar nerve coverage (UNC) method. This technique does not require anterior transposition of the ulnar nerve.

In this report, we describe three patients with distal humerus fractures who had no preoperative ulnar neuropathy, and we examine whether fixation using the UNC method prevented ulnar neuropathy with consideration of the results of postoperative nerve conduction studies.

## 2. Case Presentation

**Figure 1:**
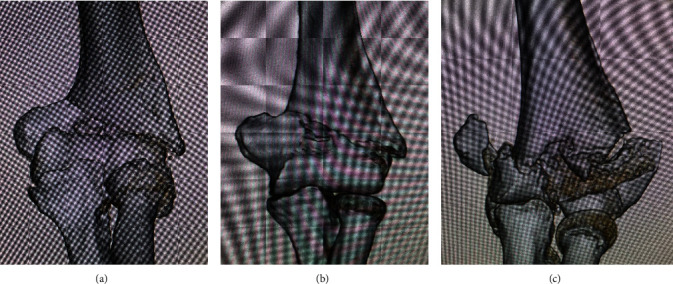
Three-dimensional computed tomography images at the time of injury: (a) patient 1; (b) patient 2; (c) patient 3.

Three patients who underwent biplate fixation for distal humerus fractures at our hospital from April to June 2022 were analyzed. Patient 1 was a 76-year-old woman with a left distal humerus fracture (AO/OTA classification [4]: 13A2.3), patient 2 was an 82-year-old woman with a left distal humerus fracture (AO/OTA classification: 13A2.3), and patient 3 was a 34-year-old woman with a left distal humerus fracture (AO/OTA classification: 13C3, combined with fractures of the olecranon and radial neck) (Figure 1). None of the patients had numbness of the little finger and intrinsic muscle weakness preoperatively and had preexisting peripheral neuropathic conditions such as diabetes mellitus. The patients were immobilized in splints on the day of injury, and fixation was performed 2 to 6 days after injury. The patients were placed in the lateral decubitus position under general anesthesia, and the fixation was performed with two plates (VA-LCP distal humerus plate; DePuy Synthes, Raynham, MA, USA), one posterolateral and one medial, via a posterior approach. In patient 3, the olecranon and triceps were reflected at the olecranon fracture to expose the joint, the articular surface was reconstructed with a poly-L-lactide pin and a headless compression screw, and an iliac bone graft was used for the bone defect of the medial column. No written consent has been obtained from the patients as there is no patient identifiable data included in this case report.

**Figure 2:**
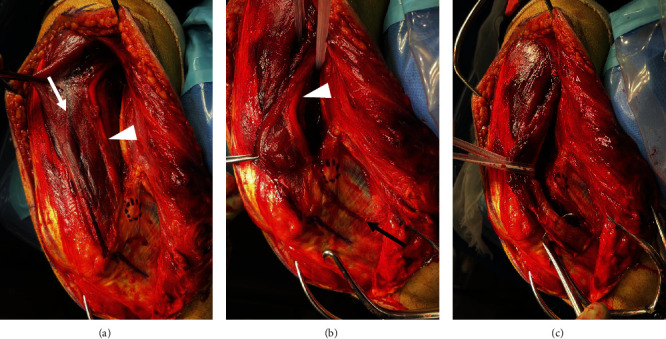
UNC method in practice (circled area is the medial epicondyle of the humerus). (a) The ulnar nerve (white arrowhead) is kept attached to the triceps brachii muscle, and the muscle is divided longitudinally about 1 cm lateral to the ulnar nerve (white arrow). (b) Distally, the Osborne ligament (black arrow) is first dissected, and the ulnar nerve is identified. Next, the ulnar head of the flexor carpi ulnaris with the ulnar nerve is elevated under the periosteum. (c) After ulnar nerve dissection with the UNC method, the ulnar nerve is approximately semicircumferentially covered by the triceps brachii and flexor carpi ulnaris muscles. The distal dissection should extend to the point where the medial plate can be applied.

**Figure 3:**
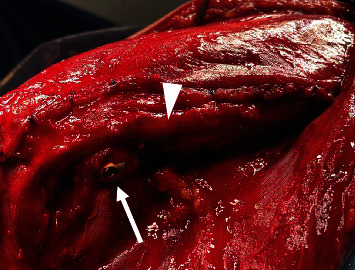
Position of the ulnar nerve and medial plate with the elbow flexed at 75 degrees. When the triceps muscles are sutured together after plate fixation, the ulnar nerve (white arrowhead) returns to its anatomical position and does not interfere with the medial plate (white arrow).

The ulnar nerve was first identified proximally, and the location of the nerve was then identified distally. At this time, the soft tissue superficial to the nerve was detached, but no dissection was performed to detach the nerve from the underlying tissue. Next, the triceps brachii was longitudinally dissected approximately 1 cm from the ulnar nerve, with the triceps still attached to the ulnar nerve (Figure 2(a)). Distal to the medial epicondyle, the Osborne ligament was first dissected to identify the ulnar nerve, and the ulnar head of the flexor carpi ulnaris muscle was then raised under the periosteum with the ulnar nerve (Figure 2(b)). Distal dissection was minimized as much as possible to prevent the ulnar nerve from interfering with the medial plate. With these maneuvers, the ulnar nerve was elevated, proximally wrapped around the triceps brachii muscle, and distally wrapped around the flexor carpi ulnaris muscle (Figure 2(c)). The ulnar nerve was taped during the operation, but forceps were not used because of the external force applied. After bony fixation, the ulnar nerve was returned to its anatomical position by suturing the divided triceps muscle and fascia of the flexor carpi ulnaris (Figure 3). The procedure was terminated after confirming that the ulnar nerve and medial plate did not interfere with each other during passive elbow movement.

We evaluated the patients for postoperative signs of ulnar neuropathy, such as numbness and muscle weakness, and nerve conduction studies were performed after more than 3 months postoperatively. The motor nerve conduction velocity (MCV) was measured from above the elbow to below the elbow, and the compound muscle action potential (CMAP) was measured as the action potential of the abductor pollicis minor muscle when the ulnar nerve was stimulated above the elbow. The sensory nerve conduction velocity (SCV) and sensory nerve action potential (SNAP) in the little finger were measured when the ulnar nerve was stimulated above the elbow.

**Figure 4:**
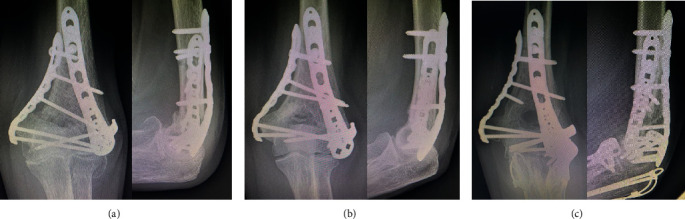
X-ray images at the final follow-up: (a) patient 1; (b) patient 2; (c) patient 3.

The postoperative follow-up period was 6 to 8 months, and all three patients achieved bony union (Figure 4). No patients experienced any numbness or muscle weakness in the ulnar nerve area during the observation period. Two of the three patients underwent the operation with both general and regional anesthesia, so sensory function and muscle strength could not be evaluated on the day of surgery. The remaining patient, who did not undergo regional anesthesia, developed postoperative restlessness and also could not be evaluated on the day of surgery. None of the patients had numbness in the ulnar nerve area the day after surgery. The results of the nerve conduction studies are shown in Table 1 (patient 1 was evaluated at 5 months postoperatively, patient 2 at 4 months, and patient 3 at 3 months). The MCV and SCV were higher than those of the noninjured side in patients 1 and 3 but lower than those of the noninjured side in patient 2 (the MCV was 83.2% and SCV was 98.6% of those on the noninjured side). Although the CMAP and SNAP were comparable or higher than those of the noninjured side in patients 1 and 3, the CMAP was 77.3% of that on the noninjured side, and the SNAP was comparable in patient 2.

## 3. Discussion

In this study, three patients with distal humerus fractures who had no preoperative ulnar neuropathy underwent plate fixation using the UNC method, and none of the three patients developed ulnar neuropathy. Although the number of cases is small and more cases are needed in the future, this study suggests that preservation of blood flow to the ulnar nerve may be an important factor in preventing postoperative ulnar neuropathy. Patients who undergo conventional ulnar nerve dissection often complain of numbness for at least several days after surgery, and the frequency of the postoperative numbness was reported up to 33.3% [5, 6]. With the UNC method, however, there was no numbness from the day after surgery, clearly demonstrating the effectiveness of this technique in preventing ulnar neuropathy.

The prevention of postoperative ulnar neuropathy has long been discussed in terms of anterior transposition of the ulnar nerve [2, 6–9]. However, the preventive effect of anterior transposition has not been clarified, and it is therefore necessary to consider the prevention of postoperative ulnar neuropathy from a different perspective. We focused on blood flow of the ulnar nerve, which has been the focus of many previous studies in this field. Nakamura et al. [10] reported that ligation of the three extrinsic vessels of the ulnar nerve (superior ulnar collateral artery, inferior ulnar collateral artery, and posterior ulnar recurrent artery from proximal to distal) reduced intranerve blood flow by up to 72%. In addition, there are reports of changes of intranerve blood flow in response to external forces (e.g., venous stasis at 8% traction and interruption at 15% traction in the rabbit tibial nerve as well as venous stasis at 20 mmHg compression and interruption at 60 mmHg compression also in the rabbit tibial nerve), indicating that peripheral nerve blood flow is extremely vulnerable to external forces [11, 12]. If the ulnar nerve is conventionally dissected bare during fixation of a distal humerus fracture, blood flow to the ulnar nerve is expected to be greatly reduced because all three extrinsic vessels will probably be dissected and exposed to external forces such as traction and compression throughout the surgery. In the herein-described UNC method, soft tissues are kept attached to the ulnar nerve to prevent loss of blood flow to the ulnar nerve; however, it is still unclear how much blood flow is actually preserved and how much external force on the nerve is prevented during surgery. Kurashige et al. [13] performed a procedure in which the ulnar nerve was not detached from the triceps muscle in 13 patients and reported that postoperative ulnar neuropathy was prevented in all patients. In terms of a method that maximally preserves blood flow to the nerve, the objective is the same as in our UNC method. However, in their method, the ulnar nerve remains attached to the triceps muscle; thus, there is a risk of damaging the nerve with triceps retraction when the medial plating is performed. In addition, their method cannot be applied when olecranon osteotomy is required. Krkovic et al. [14] reported a method of elevating the entire surrounding soft tissue, as we did. However, they stated in their report that their method was technically difficult because the nerve was not visible during the surgery, which occurred because the soft tissues superficial to the nerve were not dissected. The UNC method is not much different from conventional ulnar nerve dissection, and it is technically easier in that it does not require detailed dissection. Furthermore, because the visibility of the ulnar nerve is good, this method is considered safe.

**Figure 5:**
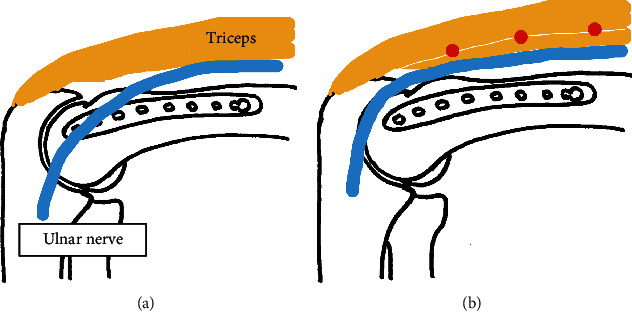
Schema of the left distal humerus. (a) When the ulnar nerve is transferred anteriorly, the ulnar nerve interferes with the plate. (b) In the UNC method, when the triceps muscles are finally sutured together (red circle), the ulnar nerve returns to its anatomical position and does not interfere with the plate.

Our UNC method does not allow anterior transposition. Ahmed et al. and Meinberg et al. [2, 6] reported a 4.0- to 4.8-fold increase in postoperative ulnar neuropathy when anterior transposition was performed during fixation of distal humerus fractures. Chander et al. [15] also reported that when nerve dissection was performed intraoperatively but without anterior transposition, only mild disability occurred in 3 of 41 patients. By contrast, Ruan et al. [16] reported better results with anterior transposition in 29 distal humerus fractures with preoperative ulnar neuropathy. However, that was a report on treatment of neuropathy and is not helpful in discussing prevention of postoperative neuropathy. We rather believe that anterior transposition is more likely to cause ulnar neuropathy because the ulnar nerve is more likely to attach to the implant (Figure 5(a)). In terms of its anatomy, the ulnar nerve runs dorsal to the medial interosseous septum in the distal humerus, passes dorsal to the medial epicondyle, and then enters the flexor carpi ulnaris muscle. The medial plate is in contact with the medial aspect of the humerus, which means that the nerve and plate are less likely to attach if the ulnar nerve is in an anatomical position. In other words, anterior transposition would make it easier for the nerve and plate to contact each other, but the reason for such a procedure is the position of the patient during surgery. Fixation of a distal humerus fracture is most often performed in the lateral or prone position [17, 18]; because the upper arm itself is upside down, if the ulnar nerve is dissected bare, it will hang anteriorly due to gravity. If the ulnar nerve is dissected bare, it cannot be sutured and is difficult to return it to its anatomical position. In other words, if the ulnar nerve is conventionally dissected, it will have to be transposed anteriorly. On the other hand, in the UNC method, the ulnar nerve can be moved backward against gravity by suturing the triceps muscles and fascia of the flexor carpi ulnaris together after plate fixation (Figure 5(b)). Even after suturing them, the ulnar nerve moved slightly anteriorly during deep flexion of the elbow as it was pushed out by the triceps muscle, but at the same time, it also moved superficially, so it did not interfere with the plate. We believe that this is advantageous in preventing neuropathy because the ulnar nerve returns to its anatomical position and is no longer attached to the plate. The method described by Moritani et al. [3], which we referred to in our study, involves final anterior transposition of the ulnar nerve, and we have improved on this point in our development of the UNC method.

Regarding the nerve conduction studies, none of the three patients had symptoms of numbness or muscle weakness. In patient 2, however, both the MCV and CMAP were somewhat lower than those on the noninjured side, suggesting that there was some damage to the ulnar nerve. The SCV was 0.8 m/s lower than that on the noninjured side, and the SNAP was the same as that on the noninjured side. The UNC method does not completely preserve blood flow to the ulnar nerve and does not completely eliminate the external force applied to the ulnar nerve during surgery, which may have influenced our results. However, the effects were only slight and not strong enough to cause symptoms, and we believe that we were able to achieve our goal of preventing ulnar neuropathy. In both patients 1 and 3, the values were higher than those of the noninjured side, leading us to conclude that there was no ulnar neuropathy caused by the surgery.

Limitations of this study include the small number of patients (three), the lack of preoperative nerve conduction studies, and the lack of objective evaluation of blood flow in the ulnar nerve. Although more cases will be needed in the future, we believe that it is difficult to perform a nerve conduction study before surgery in the presence of pain from the fracture itself. Thus, we can only refer to the values obtained on the noninjured side. Regarding objective evaluation of blood flow in the ulnar nerve, we would like to conduct further research on the use of high-resonance ultrasonography to detect the extrinsic ulnar nerve artery in the early postoperative period.

In this study, three patients with distal humerus fractures who did not have ulnar neuropathy preoperatively underwent plate fixation using the UNC method, and none of the patients developed ulnar neuropathy postoperatively. Nerve conduction studies indicated that there was minimal, if any, adverse effect on the ulnar nerve. We believe that postoperative ulnar neuropathy was prevented by preserving the soft tissue around the ulnar nerve (thereby preserving blood flow to the ulnar nerve), reducing the external force on the nerve during surgery, and finally suturing the triceps muscles and fascia of the flexor carpi ulnaris together so that the ulnar nerve returned to its anatomical position and did not interfere with the plate. The UNC method is suggested to have the potential to prevent ulnar neuropathy after fixation of distal humerus fractures with simple procedures.

## Figures and Tables

**Table 1:** Results of postoperative nerve conduction study of the ulnar nerve.

	Injured side				Noninjured side			
	MCV (m/s)	SCV (m/s)	CMAP (mV)	SNAP (uV)	MCV (m/s)	SCV (m/s)	CMAP (mV)	SNAP (uV)
Case 1	41.7	61.1	2.5	24.6	36.5	51.6	2.4	4.2
Case 2	52.8	55.4	3.4	0.8	63.4	56.2	4.4	0.8
Case 3	56	71.4	6.3	15.7	50.4	58.3	5.6	1.2

MCV: motor nerve conduction velocity; SCV: sensory nerve conduction velocity; CMAP: compound motor action potential; SNAP: sensory nerve action potential.
